# Tailoring Zinc Anode Interface with a Lard Derivative Coating for High-Performance Aqueous Batteries

**DOI:** 10.3390/ma19143009

**Published:** 2026-07-13

**Authors:** Wenqiang Xu, Shuyue Tan, Di Deng, Bingbing Hu

**Affiliations:** 1 China–Spain Collaborative Research Center for Advanced Materials, College of Materials Science and Engineering, Chongqing Jiaotong University, Chongqing 400074, China; 632308040102@mails.cqjtu.edu.cn (W.X.); 17749933708@163.com (S.T.); 2 Chongqing Huanli Circular Technology Co., Ltd., Dazu District, Chongqing 400900, China; 18306076923@163.com; 3 Institute for New Energy Materials and Equipment in Transportation, Chongqing Jiaotong University, Chongqing 400074, China

**Keywords:** aqueous zinc-ion battery, lard derivative coating, hydrophobic zincophile, dendrite inhibition, interface stabilization

## Abstract

**Highlights:**

- A lard-derived hydrophobic/zincophilic coating extends symmetric cell lifespan to >3500 h.
- Achieved 99.8% CE over 2400 cycles (Zn||Cu) and stable full-cell capacity of ~400 mAh·g^−1^ for 800 cycles at 5 A·g^−1^.
- This scalable, low-cost bionic strategy fundamentally resolves dendrite growth and water-induced side reactions.
- It meets grid-scale storage demands and offers a generalized stabilization design for other highly active metal anodes.

**Abstract:**

In order to solve the bottleneck problems of zinc anode in aqueous zinc-ion batteries, such as dendrite disorder growth, hydrogen evolution corrosion, and interface passivation, lard derivative coating (LDC) was fabricated on zinc anode using a coating–calcination process. The microstructure, surface physical, and chemical properties of LDC and its influence on zinc deposition behavior and interface stability were investigated using a combination of techniques, including scanning electron microscopy (SEM), energy-dispersive X-ray spectroscopy (EDS), X-ray diffraction (XRD), Fourier transform infrared spectroscopy (FTIR), and X-ray photoelectron spectroscopy (XPS). The LDC-modified Zn anode (LDC@Zn) delivers stable cycling for over 3500 h at 1 mA·cm^−2^/0.5 mAh·cm^−2^. In Zn||Cu asymmetric cells, an average coulombic efficiency of 99.8% is achieved over 2400 cycles, confirming highly reversible Zn plating/stripping behavior. Furthermore, the full cell maintains a reversible capacity of ~400 mAh·g^−1^ after 800 cycles at 5 A·g^−1^, demonstrating excellent rate capability and long-term stability. Overall, this work innovatively demonstrates that the LDC interphase integrates hydrophobic suppression of side reactions and zincophilic regulation of Zn^2+^ deposition within a single architecture, enabling a synergistic balance between interfacial stability and controlled ion transport, and providing a scalable strategy for stable Zn anodes and new insights into interfacial engineering.

## 1. Introduction

With the depletion of global fossil fuel and the increase of environmental problems, the development of safe, efficient and low-cost energy storage technology has become the core demand to support the large-scale application of renewable energy [1,2,3,4]. Although lithium-ion batteries (LIBs) currently dominate the energy storage market due to their high energy density and mature industrialization, their widespread application is still constrained by several intrinsic limitations. In particular, the use of flammable organic liquid electrolytes poses significant safety risks, including leakage, thermal runaway, and even combustion under abuse conditions such as overcharging or mechanical damage. In addition, the reliance on scarce and expensive materials (e.g., lithium, cobalt, and nickel) leads to relatively high production costs and raises sustainability concerns. These issues collectively hinder the long-term development of conventional lithium-ion batteries for large-scale energy storage applications [5,6,7,8]. Aqueous zinc-ion batteries (AZIBs) show great potential for clean energy transformation due to their advantages of high safety, low cost, environmental friendliness and considerable performance [9,10,11]. However, the application of zinc anodes is severely limited because zinc ions are uneven in the surface electric field distribution during deposition and are easy to form sharp zinc dendrites [12,13,14,15]. In addition, during battery operation, hydrogen evolution reaction and chemical corrosion will inevitably occur, resulting in the generation of by-products (Zn_4_SO_4_ (OH)_6_·xH_2_O, Zn(OH)_2_, ZnO), zinc surface passivation, and electrolyte performance degradation, thus affecting the deposition and transmission of zinc ions, resulting in battery capacity attenuation and coulomb efficiency reduction [16,17].

In order to address the aforementioned challenges associated with zinc metal anodes, extensive research efforts have been devoted to developing various modification strategies, including electrolyte optimization, zinc anode structural engineering, and interfacial protection layer construction. Among these approaches, constructing an artificial interfacial layer on the zinc anode surface is widely regarded as one of the most direct and effective strategies. The primary function of such interfacial layers is to physically and/or chemically isolate the zinc electrode from the aqueous electrolyte, thereby mitigating direct water-induced side reactions, regulating Zn^2+^ flux, and promoting uniform nucleation and deposition behavior to suppress dendrite growth. Accordingly, rational design of multifunctional interfacial coatings that are simultaneously stable, efficient, and environmentally benign has become increasingly important. Such coatings are expected not only to provide a physical barrier against electrolyte corrosion but also to offer additional functionalities such as Zn^2+^ affinity, ion transport regulation, and interfacial energy modulation [18,19]. Therefore, developing sustainable and multifunctional protective layers to simultaneously address dendrite formation, hydrogen evolution, and corrosion remains a critical challenge for advanced zinc-based energy storage systems.

In view of the above challenges, inspiration is drawn from the traditional “pot-opening” process in rural areas. Pot-opening is a traditional iron pan treatment process in which a new iron pan is heated at high temperature and coated with animal fat (such as lard) to form a stable oily protective layer on the surface of the metal by heat treatment, thereby achieving rust prevention and reducing surface adhesion. The essence of this simple process is to use natural oil to build a dense hydrophobic protective layer on the metal surface to block the water vapor in the air from contacting the iron surface. When it is mapped into aqueous zinc-ion battery system, a bionic intelligent in-situ interface protection strategy—lard-derived coating—can be formed. This has better thermal stability and structural compactness than some vegetable oils, so it is selected as the precursor of this study.

Specifically, conventional artificial protective coatings applied to Zn anodes are typically fabricated via coating, dip-coating, or spray-coating methods. Although these approaches can provide partial interfacial protection, they often suffer from issues such as insufficient adhesion strength to the Zn substrate and non-uniform coating thickness, which may lead to local failure during long-term cycling.

In contrast, the lard-derived coating (LDC) developed in this work is constructed through a solution coating combined with a subsequent thermal treatment process, enabling the formation of a uniform, conformal, and densely covered interfacial layer. This layer is physically anchored on the Zn surface and uniformly covers the electrode, effectively isolating it from the aqueous electrolyte while maintaining interfacial integrity during cycling. As a result, the coating serves as a stable physical barrier that regulates electrolyte contact and interfacial reactions in aqueous zinc-ion batteries.

The main components of lard consists mainly of triglycerides and long-chain fatty acids, and these triglycerides are cleaved during heat treatment to form fatty acid structures containing carboxyl groups [20]. These fatty acid molecules have both hydrophobic alkyl chains and zincophilic-COOH groups, which form a bifunctional interface layer on zinc surfaces. Among them, the long-chain alkyl groups are arranged outward to construct a hydrophobic barrier on the zinc surface, which significantly reduces the wettability of electrodes and electrolyte, hinders the penetration of free water and hydrated hydrogen ions (H_3_O^+^) to the zinc interface, and inhibits the occurrence of hydrogen evolution reaction from the source. At the same time, the hydrophobic environment effectively avoids local OH^−^ accumulation, greatly weakens the nucleation tendency of passivation byproducts such as basic zinc sulfate, and maintains chemical stability [21,22]. More importantly, the carboxyl group (-COOH) at the end of the fatty acid molecule has natural affinity coordination ability for Zn^2+^. This group is uniformly anchored on the surface of the zinc anode, which can be used as an induced nucleation site to guide the uniform distribution of Zn^2+^ flux, so that zinc deposition grows layer by layer along the two-dimensional plane instead of abnormally accumulating at the local tip [23,24,25]. This “zinc-friendly positioning” effect effectively inhibits the classical “tip discharge” and dendrite growth, and significantly improves the reversibility of deposition/stripping. The coordination of the two not only retains the advantages of firm interface combination and accurate coverage of the in-situ protective layer, but also realizes the functional leap from passive barrier to active regulation.

This work proposes a biomimetic interfacial engineering strategy based on natural fat-derived components to stabilize aqueous zinc anodes. The key innovation lies in the construction of a lard-derived interphase (LDC) that has not been previously reported for zinc anode protection. This interface simultaneously integrates multiple functional roles, including hydrophobic regulation to suppress side reactions and water-induced corrosion, as well as zincophilic coordination sites (e.g., carboxyl groups) that guide Zn^2+^ distribution and promote uniform deposition behavior. Unlike conventional passive blocking layers that simply isolate the electrode from the electrolyte, the LDC enables an active regulation of Zn deposition kinetics by coupling interfacial hydrophobicity with Zn^2+^-affinitive chemical interactions. As a result, the Zn anode exhibits significantly improved electrochemical stability, achieving an extended cycling lifespan (>3500 h) and high coulombic efficiency (99.8%) under representative conditions. This study provides a scalable strategy for constructing multifunctional interfacial layers, offering a new perspective for the rational design of stable metal anodes in aqueous energy storage systems.

## 2. Materials and Methods

### 2.1. Materials Preparation

#### 2.1.1. Preparation of LDC@Zn

**Figure 1:**
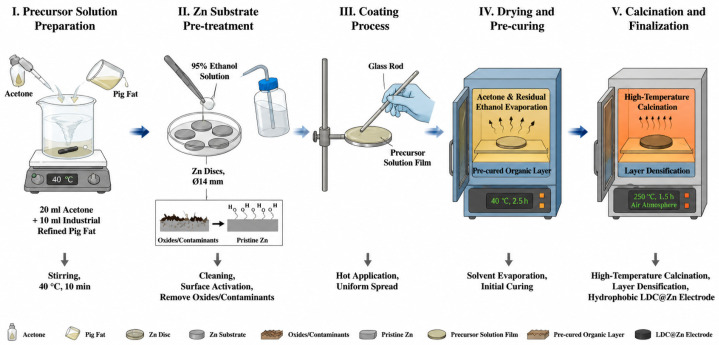
LDC@Zn Preparation Process.

LDC modified zinc anodes (LDC@Zn) were prepared as shown in Figure 1 and Figure S1. 10 mL of industrial refined lard (Fufei, Guangzhou, China) was diluted with 20 mL of 99.5% acetone (Chron, Chengdu, China) solution in a clean beaker. The mixed solution was stirred at 40 °C for 10 min. Several zinc sheets with a diameter of 14 mm were selected and washed with 95% ethanol (Chuandong, Chongqing, China) solution. A glass rod was used to dip an appropriate amount of solution and evenly coat the zinc sheet surface while hot to ensure complete coating and uniform thickness. After coating, the zinc sheet was placed in an oven and dried at 40 °C for 2.5 h. After the organic solvent is completely volatilized, the zinc sheet is transferred to a muffle furnace for high temperature calcination treatment. In the air atmosphere, it is calcined at a constant temperature of 250 °C for 1.5 h. The selection of 250 °C is critical, as it is sufficient to induce controlled thermal decomposition and rearrangement of the long-chain fatty components, leading to the formation of a stable, dense, and hydrophobic organic interfacial layer, while avoiding excessive carbonization or structural collapse of the coating. In this way, a dense and uniform hydrophobic zinc-loving organic protective layer, LDC@Zn, will be formed on the surface of the zinc sheet. As shown in Figure S2, the thickness of the LDC was estimated by stacking multiple samples to improve measurement accuracy. Specifically, six bare Zn foils exhibited a total thickness of 0.60 mm, whereas six LDC-modified Zn foils showed a total thickness of 1.40 mm. Based on these measurements, the thickness of a single LDC layer was calculated to be approximately 0.13 mm (130 μm).

#### 2.1.2. Preparation of Cathode Material

Preparation of V_2_O_3_ cathode material: Vanadium pentoxide (V_2_O_5_) powder (Aladdin, Shanghai, China) and oxalic acid dihydrate are mixed in a molar ratio of 1:2.5 and dissolved in deionized water, and continuously stirred magnetically at 60 °C for 2 h until the solution is completely converted into a dark blue clear liquid; then, the solution is transferred to a stainless steel reaction kettle, tightened, and placed in an oven for continuous hydrothermal reaction at 180 °C for 18 h; after the reaction is completed and naturally cooled to room temperature with the oven, the bottom precipitate is collected and alternately centrifuged and washed with deionized water and anhydrous ethanol (Chuandong, Chongqing, China) three times, and then the precipitate is placed in a vacuum drying oven and dried at 80 °C for 12 h to obtain the precursor. Finally, the precursor powder is evenly ground and spread in a crucible and pushed into a tubular furnace, heated to 700 °C under the protection of continuous introduction of high-purity argon and calcined for 3 h, and after it is naturally cooled to room temperature under the protection of argon, well-crystallized V_2_O_3_ can be successfully fabricated. The XRD pattern of V_2_O_3_ (Figure S3) shows that we have successfully prepared V_2_O_3_.

### 2.2. Electrochemical Measurements

Figure S4 is a schematic diagram of battery configuration. For the Zn||Zn symmetric cells, LDC-modified Zn foil (LDC@Zn) was used as both working and counter electrodes. A glass fiber separator (14 mm diameter, Olegeeino, Chongqing, China) and 2 M ZnSO_4_ aqueous solution (Sinopharm, Shanghai, China) were employed as the electrolyte. For the Zn||Cu asymmetric cells, LDC@Zn was used as the working electrode, while Cu foil served as the counter electrode. The same separator and electrolyte system were used to ensure fair comparison with symmetric cells. For the full cell configuration, LDC@Zn was used as the anode, and V_2_O_3_ was employed as the cathode material. Zn(CF_3_SO_3_)_2_ aqueous solution was used as the electrolyte. Electrochemical measurements including linear sweep voltammetry (LSV), Tafel polarization analysis, linear cyclic voltammetry (CV), chronoamperometry (CA), and electrochemical impedance spectroscopy (EIS) were carried out using a CORRTEST electrochemical workstation. Galvanostatic charge–discharge (GCD) cycling, rate performance, and long-term stability tests were conducted using a NEWARE battery testing system. The electrolyte, current collectors, electrode area, and Zn areal capacity were strictly controlled to ensure fair comparison between samples. Cycling stability, rate capability, and coulombic efficiency were evaluated under defined current densities and areal capacities [26].

### 2.3. Characterization Techniques

Scanning electron microscopy (SEM) (Zeiss Gemini Sigma 300 VP, Jena, Germany), energy dispersive X-ray spectroscopy (EDS), The material’s X-ray diffraction XRD spectrum is received by the Rigaku Ultima IV ray diffractometer. Fourier transform infrared spectroscopy (FTIR) (Thermo Fisher Scientific Nicolet iS20, Waltham, MA, USA) and X-ray photoelectron spectroscopy (XPS) measurements are obtained with Al Kα X-rays on a Thermo Scientific K-Alpha.

## 3. Results and Discussion

**Figure 2:**
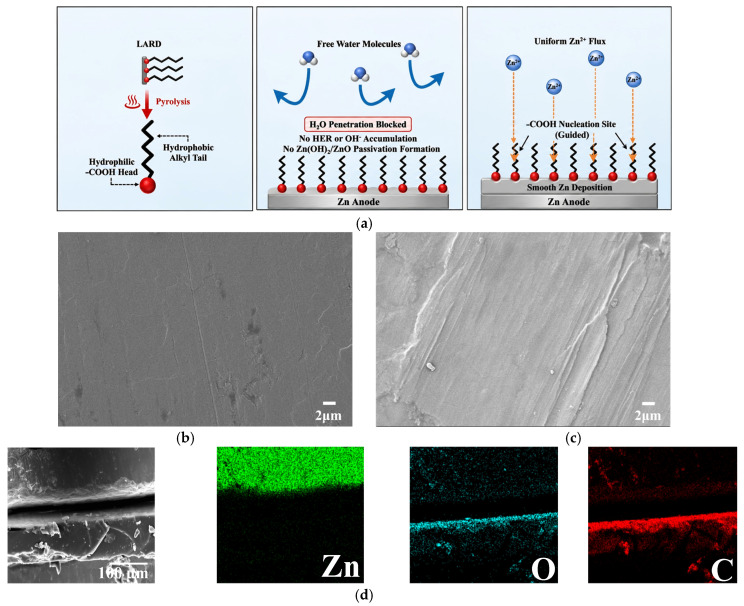
(**a**) Mechanism diagram of LDC@Zn. The SEM images of (**b**) bare Zn and (**c**) LDC@Zn in 2 μm. (**d**) Cross section SEM and EDS image of LDC@Zn.

Industrial lard was processed into a uniform coating on zinc sheets via solution application, drying, and high-temperature calcination, resulting in a dense, hydrophobic, and zincophilic protective layer. The mechanism diagram is shown in Figure 2a, the LDC interphase simultaneously provides hydrophobic protection and zincophilic regulation, which suppresses parasitic reactions while homogenizing Zn^2+^ flux and guiding uniform Zn deposition. The chain length of fatty acids plays a crucial role in determining the interfacial properties of the LDC layer. Long-chain fatty acids enhance hydrophobicity through stronger van der Waals interactions and tighter molecular packing, thereby effectively suppressing water penetration and parasitic reactions. Meanwhile, excessively long chains may increase ion transport resistance. In this work, the naturally derived lard contains fatty acids, which provides a balanced structure with sufficient hydrophobic shielding while maintaining ion transport pathways through imperfect molecular packing [27]. As shown in Figure 2b, combined with high-magnification SEM images (Figure S5a), LDC@Zn surfaces exhibit a highly smooth and dense microstructure. In contrast, the bare zinc surfaces (Figure 2c and Figure S5b) are extremely rough, with obvious mechanical scratches and grooves. This inherent unevenness easily leads to an inhomogeneous local electric field distribution, which induces a pronounced “tip effect,” where the electric field becomes highly concentrated at surface protrusions or microscopic asperities. As a result, Zn^2+^ ions preferentially migrate toward these high-field regions, leading to accelerated local deposition, continuous growth of protrusions, and ultimately the formation of disordered zinc dendrites [28,29]. In order to further verify the composition and structure of the coating, cross-section SEM and corresponding EDS elemental mapping (Figure 2d) show that C and O elements are enriched and uniformly distributed on the surface of zinc substrate, confirming the successful construction of LDC and its close combination with the substrate, effectively filling the original surface defects.

**Figure 3:**
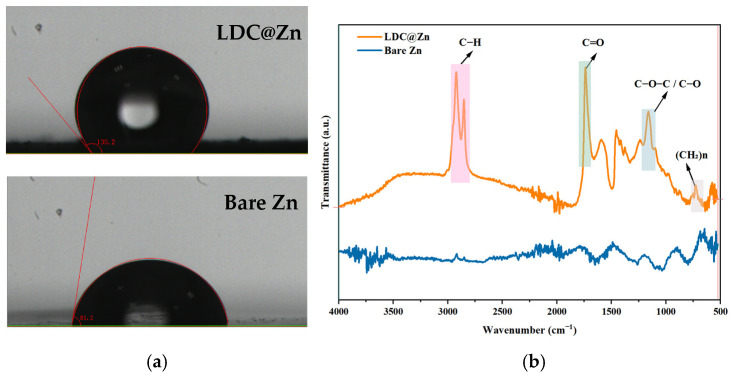
Surface physicochemical characterizations. (**a**) Contact angles of the electrolyte on the bare Zn and LDC@Zn surfaces. (**b**) FTIR spectra of bare Zn and LDC@Zn.

In order to further investigate the physicochemical properties of the in-situ interface, we first performed a contact angle test. As shown in Figure 3a, contact angle measurement is carried out by static drop method on contact angle measuring instrument. During the test, a certain volume of distilled water is added to the surface of bare zinc and LDC@Zn electrode at room temperature. After the liquid drop stabilizes, static contact angle measurement is carried out, and image fitting and angle calculation are carried out by instrument supporting software. To ensure the reliability of the results, each sample is tested at least three times at different positions, and the average value is taken. The electrolyte contact angle of the bare zinc surface is 81.2°, showing hydrophilicity, which leads to direct contact of water molecules with the zinc surface and initiates severe hydrogen evolution and corrosion side reactions. In contrast, after coating with LDC, the contact angle is greatly increased to 130.2°, demonstrating that the coating imparts excellent hydrophobicity to the electrode. This strong hydrophobic barrier can effectively block the penetration of free water into the substrate and reduce hydrogen evolution reaction and corrosion side reaction. Meanwhile, this hydrophobic property combined with the uniform deposition behavior of Zn^2+^ promoted interface stability and electrochemical performance improvement.

The chemical composition of the coating was characterized by Fourier transform infrared spectroscopy (FTIR) (Figure 3b). The LDC@Zn spectrum showed distinct peaks characteristic of long-chain alkyl groups (C–H stretching vibration of 2800–3000 cm^−1^ and (CH_2_)n in-plane rocking vibration of ~720 cm^−1^), which is the structural basis for giving the coating strong hydrophobicity. Meanwhile, the absorption peaks at ~1700 cm^−1^ and ~1100–1300 cm^−1^ are attributed to C=O and C–O/C–O–C bonds, respectively, which are consistent with those reported for long-chain fatty acid-based organic coatings in previous studies. These oxygen-containing polar groups are expected to provide uniform coordination nucleation sites for Zn^2+^ [30].

**Figure 4:**
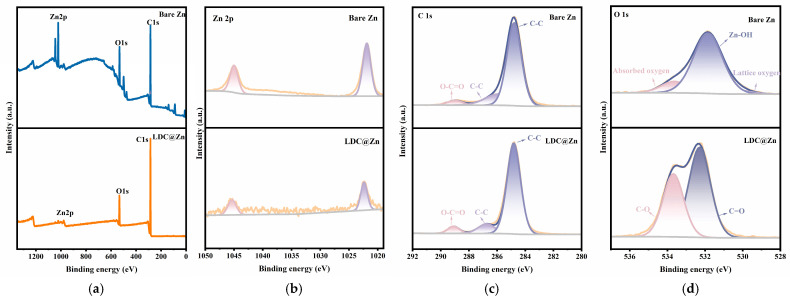
(**a**) XPS survey spectra of bare Zn and LDC@Zn. High-resolution XPS spectra of (**b**) Zn 2p, (**c**) C 1s, and (**d**) O 1s for the bare Zn and LDC@Zn electrodes.

X-ray photoelectron spectroscopy (XPS) further reveals the chemical state of the surface. XPS spectra (Figure 4a) show a significant increase in C content and a significant decrease in Zn signal on LDC@Zn surfaces compared to bare zinc. This is also confirmed by high-resolution Zn2p spectroscopy (Figure 4b), where strong attenuation of Zn signal indicates that LDC has sufficient thickness and completely covers the zinc substrate. In the high-resolution C1s (Figure 4c) and O1s (Figure 4d) spectra, O1s on the bare zinc surface is mainly composed of Zn–OH and lattice oxygen, indicating that natural oxidation and passivation phenomena inevitably exist on the original zinc surface. On the LDC@Zn surface, C=O and C–O bonds characteristic of lipid derivatives become dominant.

**Figure 5:**
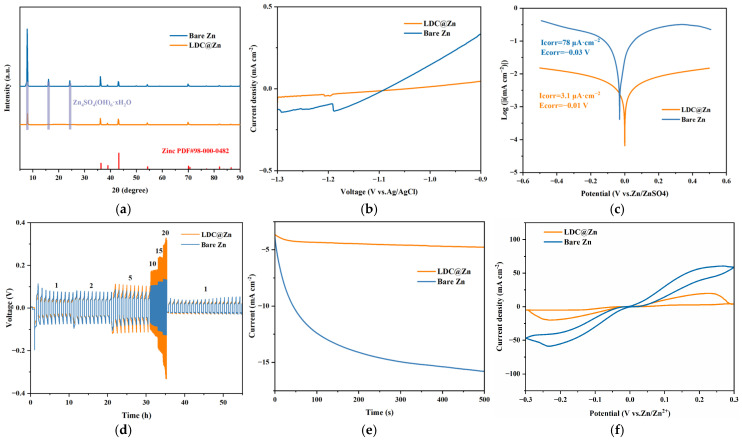
(**a**) XRD patterns of the bare Zn and LDC@Zn electrodes after immersion in 2 M ZnSO_4_ electrolyte for 10 days. (**b**) Linear sweep voltammetry (LSV) curves showing the hydrogen evolution reaction (HER). (**c**) Tafel polarization curves of the bare Zn and LDC@Zn electrodes. (**d**) Rate capability of the symmetric cells at step-increased current densities from 1 to 20 mA·cm^−2^. (**e**) Chronoamperometry (CA) curves of the bare Zn and LDC@Zn electrodes at a constant overpotential. (**f**) Cyclic voltammetry (CV) curves of the symmetric cells.

In order to fully evaluate the inhibition ability of in-situ LDC on side reactions and the regulation of deposition kinetics, we carried out a series of systematic electrochemical and structural analyses. First, the static and dynamic stability were investigated by XRD. As shown in Figure 5a, after static immersion in electrolyte for 10 days, the surface of bare zinc showed strong by-product peaks of basic zinc sulfate (ZHS), while LDC@Zn only showed pure Zn phase. More importantly, after severe cycling (Figure S6), the surface of bare zinc still forms a severe passivation layer, while LDC@Zn maintains the purity of the interface. This dynamic and static structural evolution strongly proves that the hydrophobic network of LDC effectively blocks free water, inhibiting chemical corrosion and electrochemical passivation from the source. The structural stability was precisely quantified in electrochemical tests. LSV (Figure 5b) shows that bare zinc has a sharp increase in current at negative potential, while LDC@Zn maintains an extremely flat and suppressed current response even at −1.3 V, demonstrating that the hydrophobic layer effectively hinders water decomposition. In addition, the Tafel curve (Figure 5c) accurately quantifies the corrosion resistance. The corrosion potential of LDC@Zn moved positively to −0.01 V (−0.03 V for bare zinc), and more significantly, its corrosion current density dropped sharply to 3.1 μA·cm^−2^, which was more than 25 times lower than that of bare zinc (78 μA·cm^−2^), confirming that the self-corrosion reaction was greatly slowed down.

LDC@Zn performed exceptionally well at rates ranging from 1 to 20 mA cm^−2^ (Figure 5d). It remained stable even at the limit of 20 mA·cm^−2^, whereas bare zinc exhibited violent voltage fluctuations and large polarization at this stage, which was close to short-circuiting. When the current returned to 1 mA·cm^−2^, LDC@Zn perfectly recovered to its initial state, demonstrating the strong adhesion and dynamic stability of the in-situ interface layer. With the current density increasing from 1 to 20 mAcm^−2^, the overpotential of the electrode system increases gradually, which accords with the electrochemical kinetics law; that is, the Zn^2+^ transport and nucleation processes at the interface are more restricted at high current density. It is worth noting that LDC@Zn still exhibits a relatively more controllable potential response than bare Zn in the whole current range, indicating that the interface structure can maintain stable Zn^2+^ transport and deposition behavior at high current density. In addition to suppressing side reactions, LDC can also deeply regulate the nucleation and growth kinetics of zinc. CA curve (Figure 5e) reveals the nucleation mode: at constant overpotential, the response current of bare zinc continuously increases to ~−15 mA·cm^−2^ within 500 s, which is typical of disordered 3D dendrite growth leading to increasing surface area. In contrast, LDC@Zn current stabilizes at a low level (~−4.5 mA cm^−2^) in just a few seconds, indicating limited 2D lateral diffusion and smooth nucleation. As shown in Figure 5f, compared with bare Zn, LDC@Zn exhibits a reduced current response and a more stable and controlled electrochemical behavior within the same potential window, indicating improved interfacial regulation.

**Figure 6:**
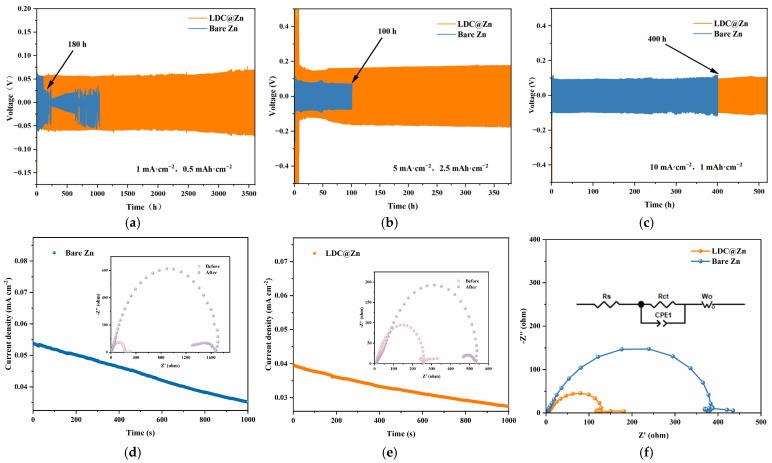
Electrochemical performance and kinetics of the symmetric cells. Galvanostatic cycling performance of the symmetric cells using bare Zn and LDC@Zn electrodes at (**a**) 1 mA cm^−2^ for 0.5 mAh·cm^−2^, (**b**) 5 mA·cm^−2^ for 2.5 mAh·cm^−2^, and (**c**) 10 mA·cm^−2^ for 1 mAh·cm^−2^. (**d**) Chronoamperometry curve and the corresponding electrochemical impedance spectroscopy (EIS) spectra before and after polarization (inset) of the bare Zn symmetric cell for the determination of the Zn^2+^ transference number. (**e**) Chronoamperometry curve and the corresponding electrochemical impedance spectroscopy (EIS) spectra before and after polarization (inset) of the LDC@Zn symmetric cell for the determination of the Zn^2+^ transference number. (**f**) EIS plots of the symmetric cells with bare Zn and LDC@Zn electrodes.

In order to systematically evaluate the long-term reversibility of the electrode in practical applications, constant current cycling tests of symmetric cells were carried out over a wide range of current densities. As shown in Figure 6a–c, bare zinc symmetric cells generally exhibit severe voltage fluctuations and premature failure at successive cycles of 1, 5 and 10 mA·cm^−2^, with lifetimes of only about 180, 100, and 400 h, respectively, typical of internal short circuits caused by disordered dendrite piercing. Remarkably, LDC@Zn symmetric cells exhibit excellent cycle stability and extremely smooth voltage curves, achieving long lifetimes of over 3500, 400, and 500 h under the corresponding conditions, respectively. LDC@Zn exhibits a more stable and continuous voltage curve, indicating that it can effectively suppress local current concentration, thus maintaining long-term stable operation. Even more striking is the catastrophic failure of bare zinc cells at approximately 300 h, even under extreme operating conditions of 20 mA·cm^−2^ (Figure S7), whereas LDC@Zn cells maintain an extremely smooth voltage hysteresis over 1000 h. This strong contrast strongly demonstrates that the in-situ protective layer is extremely robust and can effectively withstand large Zn^2+^ fluxes and drastic volume changes without mechanical degradation.

In addition, we evaluated the interfacial ion transport kinetics. We determined the Zn^2+^ transport number tZn^2+^ by chronoamperometry combined with EIS before and after polarization at 20 mV potentiostatic polarization for 1000 s. This parameter was calculated based on the Bruce–Vincent–Evans equation:

$$t_{Zn^{2+}}=\frac{I_{s}(\Delta V\;-\;I_{0}R_{0})}{I_{0}(\Delta V\;-\;I_{s}R_{s})}$$

where ΔV is the applied polarization voltage (20 mV), $I_{0}$ and $I_{s}$ are the initial and steady state currents, and $I_{0}$ and $I_{s}$ represent the interfacial charge transfer impedance before and after polarization, respectively. For the bare zinc cell (Figure 6d), the response current decays continuously within 1000 s, and the R_S_ after polarization increases sharply to about 1600 Ω, revealing slow Zn^2+^ diffusion and severe concentration polarization. In contrast, the LDC@Zn cell (Figure 6e) current stabilizes rapidly. Specific data are shown in Table 1. Based on this (Figure S8), LDC@Zn achieved a greatly increased migration number (0.69) compared to bare zinc (0.65). This indicates that dense hydrophobic chains limit the free migration of anions and free water. This inhibition of concentration polarization mitigates depletion of the space charge layer, thus delaying dendrite initiation and unlocking the ultra-long cycle life [31,32,33]. This is also confirmed by AC impedance testing (EIS, Figure 6f). Electrochemical impedance spectroscopy (EIS) measurements were carried out in symmetric Zn||Zn cells using 2 M ZnSO_4_ aqueous electrolyte. The LDC interphase does not eliminate the intrinsic Zn nucleation barrier, but instead modulates Zn^2+^ transport and interfacial deposition behavior, leading to more regulated ion flux and improved long-term cycling stability. Notably, the charge transfer resistance (Rct) of the LDC-modified Zn electrode is significantly reduced to 131.9 Ω compared with 402.3 Ω for bare Zn, indicating accelerated interfacial charge transfer kinetics and improved electrode/electrolyte interfacial compatibility, despite a relatively higher initial interfacial overpotential. LDC performs better as a composite interface layer after heat treatment than single fatty acid systems, thus verifying its bifunctional synergy.

**Figure 7:**
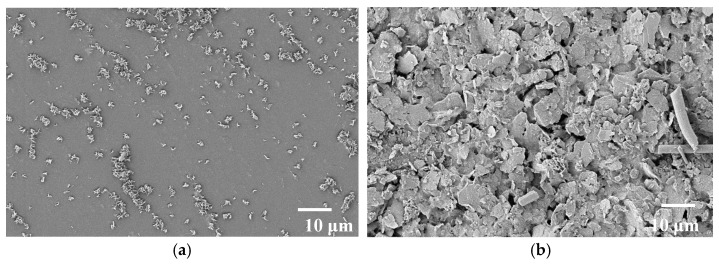

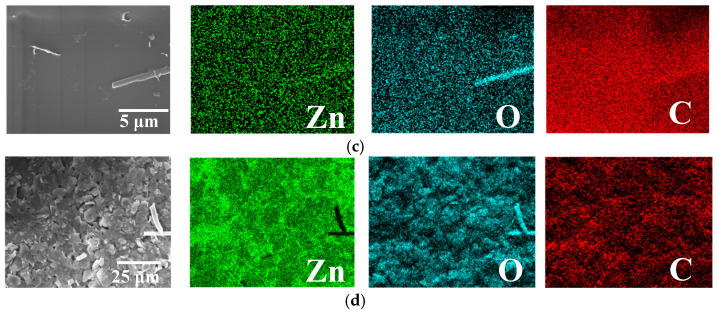
Morphological and elemental characterizations of the electrodes after continuous cycling at 1 mA·cm^−2^ for 0.5 mAh·cm^−2^. Top-view SEM images of the (**a**) LDC@Zn and (**b**) bare Zn anodes. Corresponding energy-dispersive X-ray spectroscopy (EDS) elemental mapping images of the (**c**) LDC@Zn and (**d**) bare Zn electrodes, showing the distribution of Zn, O, and C elements.

In order to visually elucidate the source of excellent electrochemical stability, the surface morphology and elemental distribution of the electrode after cycling were first characterized. After continuous cycling at 1 mA·cm^−2^ and 0.5 mAh·cm^−2^, LDC@Zn anode (Figure 7a) exhibited an exceptionally smooth and dense morphology without any prominent dendrites or insulating byproducts. In sharp contrast, the cycled bare zinc anode (Figure 7b) exhibits a completely pulverized surface, scattered with chaotic dendrites and heavy, random glassy passivation flakes. The corresponding EDS spectrum of LDC@Zn (Figure 7c) shows an extremely uniform and continuous distribution of O and C elements throughout the plane, which vividly indicates that the in-situ LDC is mechanically robust and can withstand severe volume fluctuations for long periods of time without shedding or cracking. In contrast, the highly concentrated distribution of O on bare zinc (Figure 7d) confirms the heavy passivation layer of basic zinc sulfate and the heavy accumulation of dead zinc, which inevitably blocks ion transport and initiates short circuits.

This robust dynamic structural integrity is fundamentally due to its excellent static corrosion resistance. After 10 days of immersion in 2 M ZnSO_4_ electrolyte, the bare zinc surface (Figure S9a) was severely corroded and covered with a large array of frantically growing hexagonal and petal-like lamellae. However, LDC@Zn electrode (Figure S9b) maintained an extremely clean and flat surface without any detectable byproducts. The EDS elemental spectrum corresponding to bare zinc (Figure S10a) shows that the O element signal is very strong and highly concentrated on these microstructures, visually confirming the severe ZSH formation induced by bulk water. In contrast, the extremely uniform distribution of Zn, O, and C elements on LDC@Zn (Figure S10b) confirms that the hydrophobic LDC layer effectively blocks the penetration of water and protects the active zinc from chemical corrosion. Post-cycling SEM and elemental mapping results confirm that the LDC interfacial layer remains partially preserved after long-term cycling, indicating its structural robustness and stable interfacial coverage during repeated Zn deposition and stripping processes.

**Figure 8:**
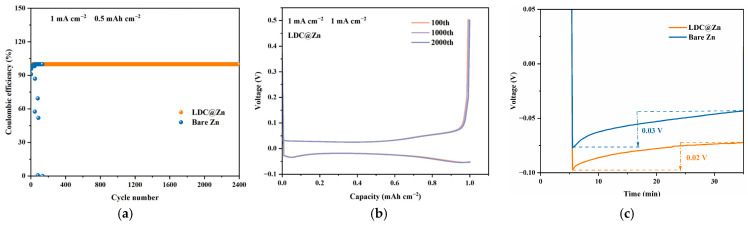
Reversibility and kinetics of Zn plating/stripping in Zn||Cu asymmetric cells. (**a**) Coulombic efficiency (CE) of the bare Zn and LDC@Zn cells at 1 mA·cm^−2^ with a capacity of 0.5 mAh·cm^−2^. (**b**) Voltage–capacity profiles of the LDC@Zn cell at the 100th, 1000th, and 2000th cycles at 1 mA·cm^−2^ and 1 mAh·cm^−2^. (**c**) Voltage–time profiles showing the initial nucleation overpotentials.

In order to fully evaluate the reversibility of zinc deposition/stripping and the inhibition of “dead zinc”, we assembled Zn||Cu asymmetric cells. As shown in Figure 8a, the coulomb efficiency of bare zinc cells fluctuated sharply at 1 mA·cm^−2^ and 0.5 mAh·cm^−2^ and dropped sharply to zero in just 150 cycles. This rapid failure was attributed to severe accumulation of dead zinc and continuous consumption of active metal zinc by side reactions. Amazingly, LDC@Zn cells exhibit an ultra-stable coulomb efficiency of about 99.8% for cycles up to 2400 cycles without any signs of decay, which strongly indicates that the in-situ hydrophobic layer excellently ensures a highly reversible zinc deposition/stripping process.

This excellent reversibility is further confirmed by the corresponding voltage–capacity curves. For bare zinc cells (Figure S11), the voltage hysteresis widens significantly at cycles 1000 and 2000, and the stripping voltage spikes abruptly, revealing severe interface deterioration and large polarization caused by insulation byproducts. In sharp contrast, LDC@Zn cells (Figure 8b) have perfectly coincident voltage curves at cycles 100, 1000, and 2000, with extremely low polarization. This perfect coincidence confirms that the LDC layer maintains an intact and highly stable interface even after very long cycles.

Cyclic voltammetry (CV, Figure S12) showed that LDC@Zn cells exhibited a negative-shifted cross potential (−0.140 V) compared to bare zinc (−0.072 V), resulting in a larger absolute nucleation overpotential difference. This increased energy barrier means that the LDC effectively limits spontaneous disordered 3D growth of dendrites, forcing Zn^2+^ to overcome a higher initial energy penalty to form fine and uniform nuclei [34,35]. Furthermore, the voltage–time curve (Figure 8c) reveals a voltage difference between the tip (initial nucleation) and the plateau (subsequent mass transfer). LDC@Zn exhibits a higher energy threshold than bare zinc (0.03 V) smaller voltage dip difference (0.02 V). This indicates that once the initial thermodynamic barrier is overcome, the zincophilic functional group in the LDC layer significantly reduces the subsequent kinetic barrier, enabling smoother, continuous, and stable 2D growth of zinc on the substrate [36,37,38,39].

**Figure 9:**
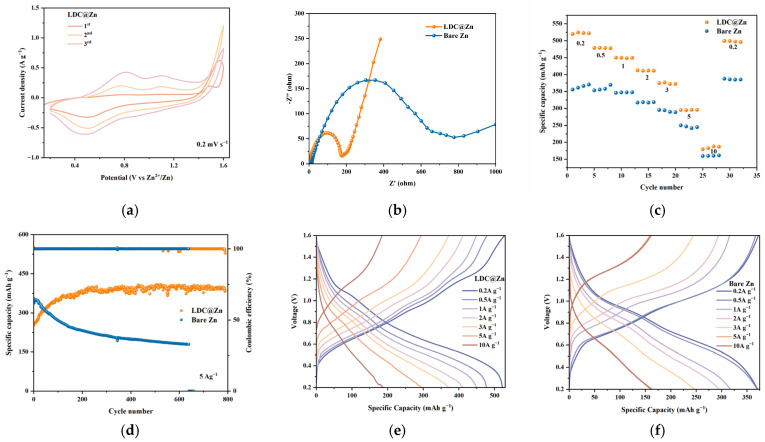
Electrochemical performance of the Zn full cells. (**a**) Cyclic voltammetry (CV) curves of the LDC@Zn full cell for the initial three cycles at 0.2 mV·s^−1^. (**b**) EIS plots of the full cells with bare Zn and LDC@Zn anodes. (**c**) Rate capability of the full cells at various current densities from 0.2 to 10 A·g^−1^. (**d**) Long-term galvanostatic cycling performance of the full cells at 5 A·g^−1^. Corresponding galvanostatic charge–discharge (GCD) profiles of the (**e**) LDC@Zn and (**f**) bare Zn full cells at different current densities.

In order to comprehensively evaluate the practical feasibility of the LDC@Zn anode, we assembled a full cell. First, the electrochemical reversibility was investigated by cyclic voltammetry (CV). As shown in Figure S13, the bare zinc full cell showed a large and abnormal irreversible oxidation peak at the first circle of high potential, revealing a serious parasitic reaction caused by the highly active bare zinc interface (e.g., water decomposition and formation of insulating by-products). Thus, the corresponding first cycle charge-discharge curve for bare zinc cells (Figure S14a) exhibits an excessive charging plateau and very low coulomb efficiency. In sharp contrast, the CV curve for LDC@Zn cells (Figure 9a) shows well-defined and highly reversible redox peaks, with cycles 2 and 3 almost completely coinciding. The highly reversible first cycle charge-discharge curves of LDC@Zn conclusively demonstrate that hydrophobic LDC effectively isolate free water and suppress initial parasitic side reactions. The excellent reversibility is also accompanied by accelerated reaction kinetics. AC impedance measurements (EIS, Figure 9b) show that the charge transfer impedance of LDC@Zn full cells is significantly reduced compared to bare zinc cells. This indicates that zincophilic groups in the LDC layer effectively promote the desolvation of Zn^2+^ and rapid charge transfer, completely overcoming the slow kinetics normally caused by severe byproduct accumulation on bare zinc surfaces [40,41,42]. Full-cell LDC@Zn exhibits excellent rate performance driven by a robust interface and fast kinetics (Figure 9c). It provides excellent specific capacities of approximately 520, 460, 410, 370, and 290 mAh·g^−1^ at current densities of 0.2, 0.5, 1, 2, and 5 A·g^−1^, respectively. Even at an ultra-high rate of 10 A·g^−1^, it maintains an appreciable capacity of approximately 180 mAh·g^−1^. In addition, when the current density recovered to 0.2 A·g^−1^, the capacity recovered perfectly. On the contrary, the capacity of bare zinc cells was significantly poor at all rates. LDC increases the initial interfacial nucleation barrier, but improves long-term Zn plating reversibility and utilization efficiency, which governs full-cell rate performance. Furthermore, the long-term cycling stability of the cell was evaluated at a high current density of 5 A g^−1^ (Figure 9d). The initial capacity reflects a pre-conditioned steady state rather than a pristine electrode state, ensuring meaningful comparison of long-term cycling behavior. The bare zinc cell experienced a sustained capacity decay to less than 180 mAh·g^−1^ due to continuous depletion of active zinc and accumulation of heavy passivation layers. Excitingly, LDC@Zn full cells achieved excellent long-term durability after undergoing the initial activation process typical of organic cathodes, stably maintaining a high reversible capacity of about 400 mAh·g^−1^ over 800 cycles and a coulomb efficiency close to 100%. Capacity retention was calculated based on the ratio of the discharge capacity at a given cycle (Cn) to the initial discharge capacity (C0), i.e., Cn/C0 × 100%. The LDC@Zn full cell maintains a high-capacity retention of approximately 95% after 800 cycles, whereas the bare Zn cell retains only ~55% of its initial capacity, confirming the significantly improved long-term cycling stability enabled by the LDC interphase. This advantage is also evident in the charge-discharge curves (Figure 9e,f and Figure S14b), where LDC@Zn cells consistently maintain significantly lower voltage polarization at various current densities compared to fast-decaying bare zinc cells. This extraordinary full-cell performance fully confirms that the in-situ LDC effectively solves the severe interfacial instability of zinc anodes, unlocking its great potential in practical high-performance aqueous batteries.

## 4. Conclusions

In summary, a green, low-cost, and scalable lard derivative coating (LDC) was successfully constructed on the Zn anode surface via a facile coating–calcining strategy. Unlike conventional artificial coatings that mainly rely on passive physical blocking or single-function regulation, serving as a robust “hydrophobic–zincophilic” dual-functional interphase, the LDC layer fundamentally shields the zinc substrate from water-induced side reactions (e.g., HER and corrosion) while actively regulating the uniform 2D deposition of Zn^2+^. Consequently, the LDC@Zn symmetric cells deliver an unprecedented ultra-long lifespan of over 3500 h at 1 mA cm^−2^, representing a significant improvement compared to bare zinc. The highly reversible plating/stripping behavior is further confirmed by a remarkable average coulombic efficiency of 99.8% over 2400 cycles in Zn||Cu asymmetric cells. Furthermore, when applied in full cells, the modified anode ensures a superior reversible capacity of ~400 mAh·g^−1^ even after 800 cycles at a high current density of 5 A·g^−1^. Looking forward, this sustainable bionic interface engineering paradigm not only accelerates the industrialization of aqueous zinc-ion batteries but also provides a universal and highly promising pathway for the stabilization design of various other highly active metal anodes. From a practical perspective, although the proposed strategy is simple and cost-effective, several challenges remain for large-scale application, including the variability of natural lard-derived precursors, precise control of coating thickness and uniformity in large-area electrodes, and optimization of thermal treatment processes for industrial scalability. Future efforts should focus on developing standardized bio-derived feedstocks, adapting roll-to-roll or spray-coating processes, and integrating low-temperature or continuous thermal treatment routes to enable scalable manufacturing.

## Figures and Tables

**Table 1:** The summary of the information of the EIS–chronoamperometry results.

Sample	Initial Current (mA cm^−2^)	Current at 500 s (mA cm^−2^)	Current at 1000 s (mA cm^−2^)	Decay Trend
Bare Zn	0.054	0.044	0.035	Fast decay
LDC@Zn	0.040	0.032	0.027	Slow, stable decay

## Data Availability

The original contributions presented in this study are included in the article/Supplementary Materials. Further inquiries can be directed to the corresponding author.

## Supplementary Materials

The following supporting information can be downloaded at: https://www.mdpi.com/article/10.3390/ma19143009/s1, Figure S1. Digital photo of bare zinc, LDC@Zn. Figure S2. Determination of LDC thickness using a stacking-based measurement approach. Figure S3. XRD patterns of V_2_O_3_. Figure S4. A schematic diagram of battery configuration. Figure S5. The SEM images of (a) LDC@Zn and (b) bare zinc in 1 μm. Figure S6. XRD patterns of LDC@Zn after cycling at 20 mA·cm^−2^/1 mAh·cm^−2^ for 1000 h. Figure S7. Cycling curves of LDC@Zn at 20 mA·cm^−2^/1 mAh·cm^−2^. Figure S8. Zinc-ion transfer numbers of LDC@Zn and bare Zn. Figure S9. Top-view SEM images of the (a) bare Zn and (b) LDC@Zn electrodes after immersion in 2 M ZnSO_4_ electrolyte for 10 days. Figure S10. Corresponding EDS elemental mapping images of the (a) bare Zn and (b) LDC@Zn electrodes after immersion for 10 days. Figure S11. Voltage–capacity profiles of the bare Zn cell at the 100th, 1000th, and 2000th cycles at 1 mA cm^−2^ and 1 mAh cm^−2^. Figure S12. Cyclic voltammetry (CV) curves of the asymmetric cells. Figure S13. CV curves of the bare Zn full cell for the initial three cycles at 0.2 mV s^−1^. Figure S14. GCD profiles of the full cells with bare Zn and LDC@Zn anodes at the (a) 1st cycle and (b) 10th cycle at 0.2 A g^−1^.

## Abbreviations

The following abbreviations are used in this manuscript:

LDC	Lard derivative coating
AZIBs	Aqueous zinc-ion batteries
LDC@Zn	LDC modified zinc anodes
