# Supplementary material for: Tailoring Zinc Anode Interface with a Lard Derivative Coating for High-Performance Aqueous Batteries
Source: Materials (Basel). 2026 Jul 13;19(14):3009. doi: 10.3390/ma19143009 (PMC13412483; doi:10.3390/ma19143009)
Supplement: Supplementary file 1 [file materials-19-03009-s001.zip › materials-4384777-supplementary.pdf]

Supporting Information

Tailoring Zinc Anode Interface with a Lard Derivative Coating for High-Performance Aqueous Batteries

Wenqiang Xu [1], Shuyue Tan [1], Di Deng [2] and Bingbing Hu [1,3,*]

[1] China–Spain Collaborative Research Center for Advanced Materials, College of Materials Science and Engineering, Chongqing Jiaotong University, Chongqing 400074, China; 632308040102@mails.cqjtu.edu.cn (W.X.); 17749933708@163.com (S.T.)

[2] Chongqing Huanli Circular Technology Co., Ltd., Dazu District, Chongqing 400900, China; 18306076923@163.com

[3] Institute for New Energy Materials and Equipment in Transportation, Chongqing Jiaotong University, Chongqing 400074, China

* Correspondence: hubingbing@cqjtu.edu.cn

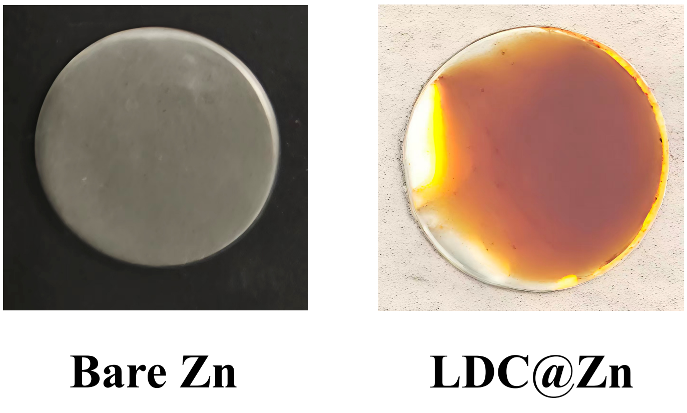

Figure S1. Digital photo of bare zinc, LDC@Zn.

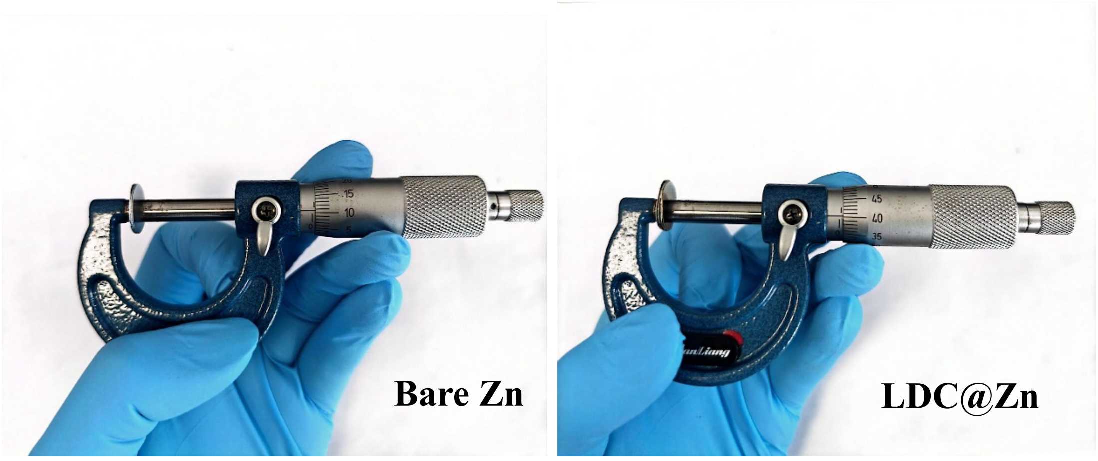

Figure S2. Determination of LDC thickness using a stacking-based measurement approach.

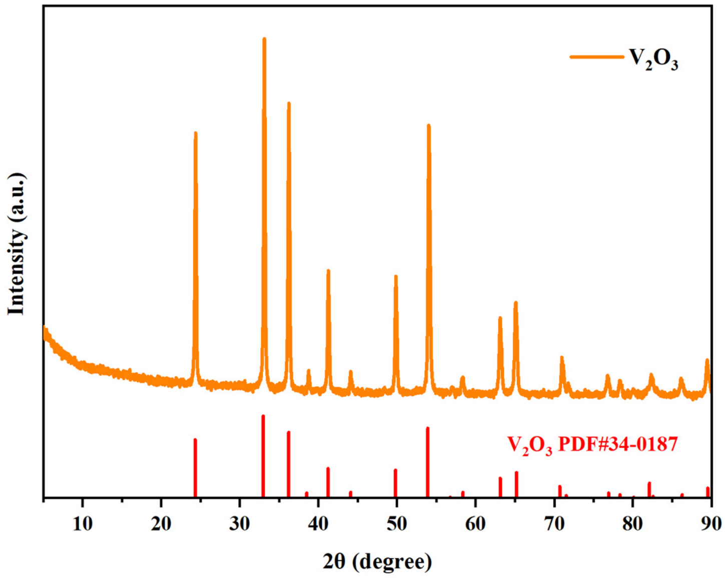

Figure S3. XRD patterns of V_2O_3.

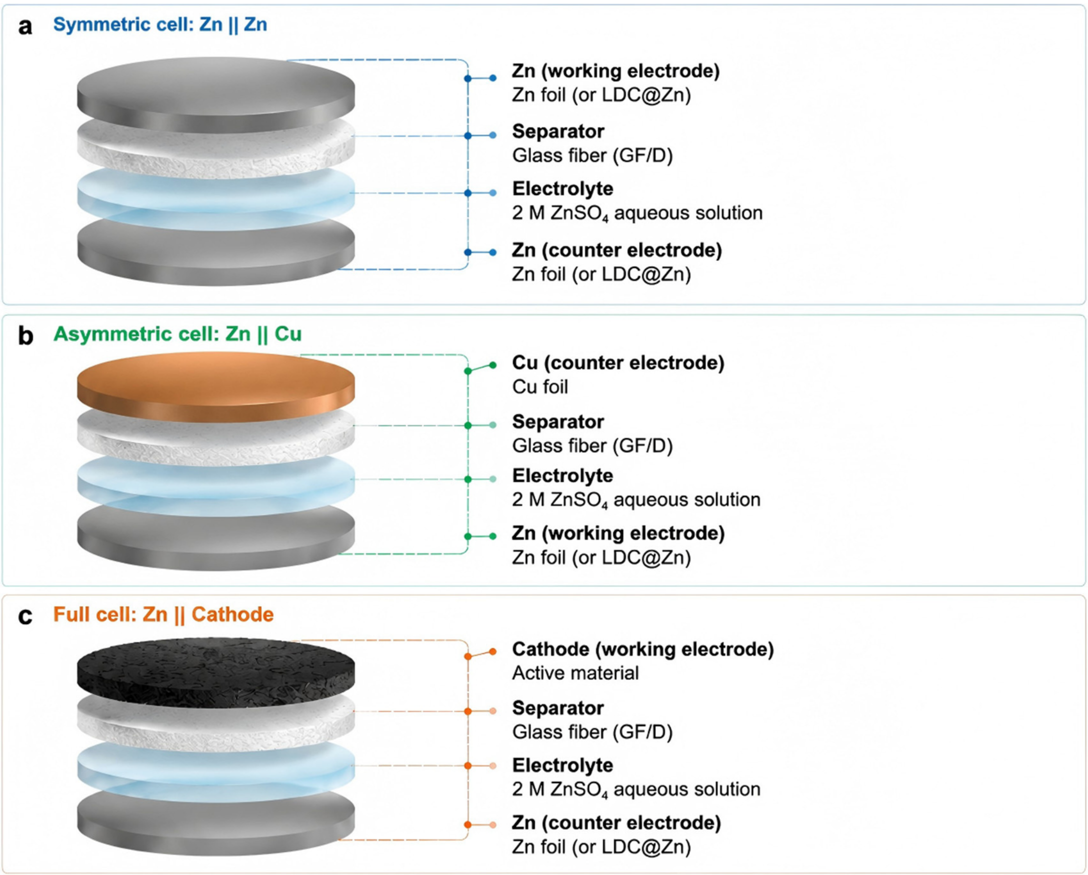

Figure S4. A schematic diagram of battery configuration.

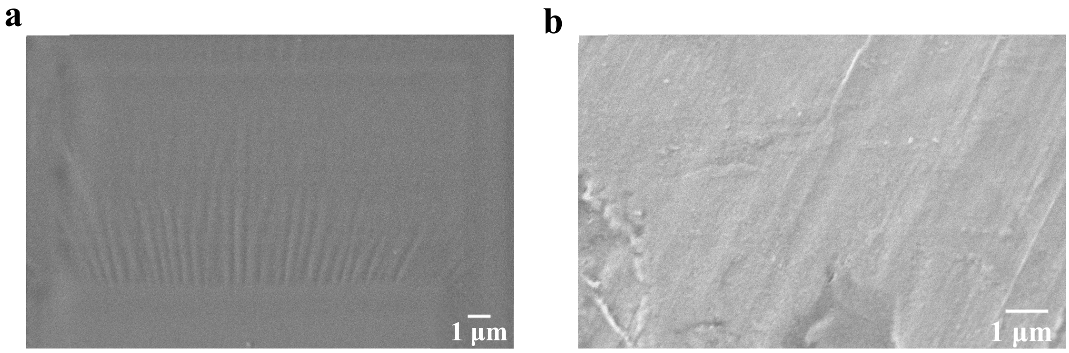

Figure S5. The SEM images of (a) LDC@Zn and (b) bare zinc in 1μm.

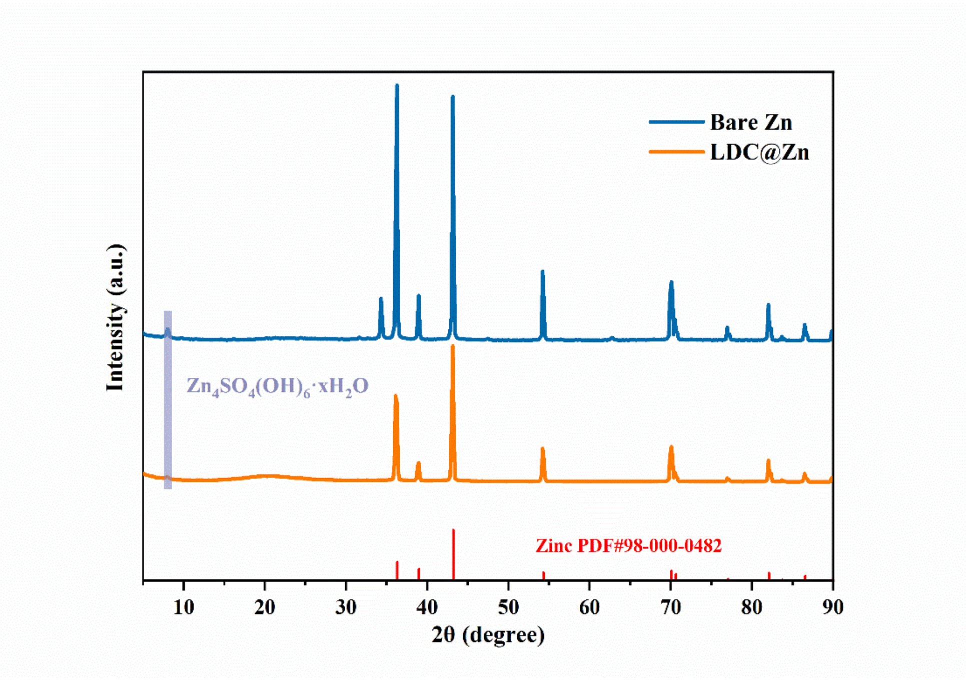

Figure S6. XRD patterns of LDC@Zn after cycling at 20 mA·cm^{-2}/1 mAh·cm^{-2} for 1000h.

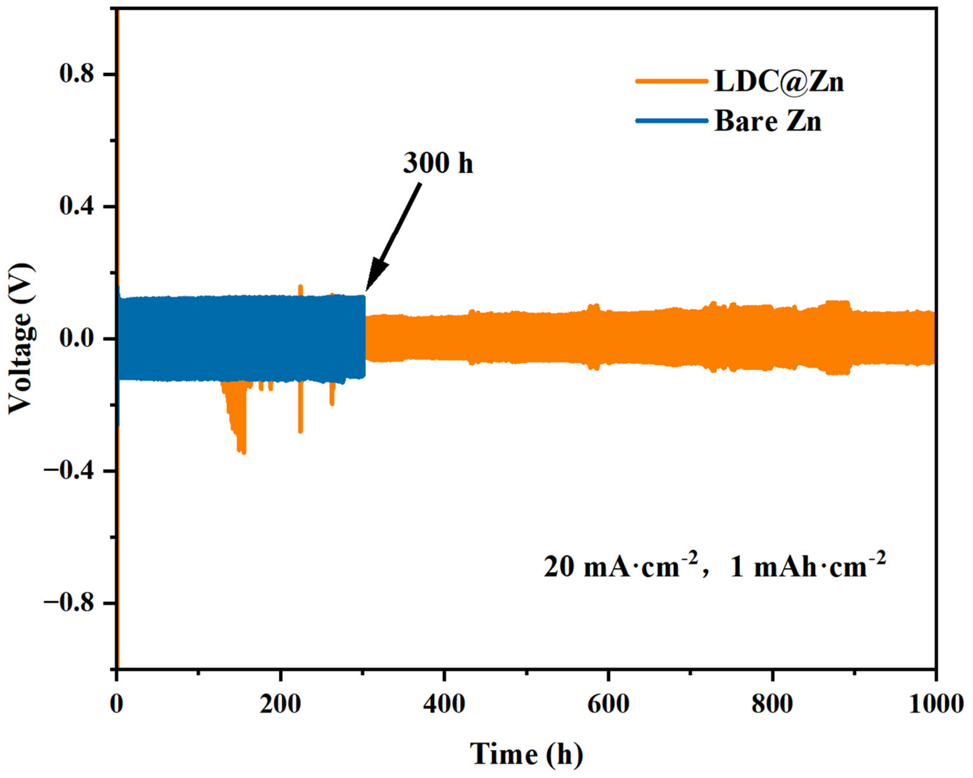

Figure S7. Cycling curves of LDC@Zn at 20 mA·cm^{-2}/1 mAh·cm^{-2}.

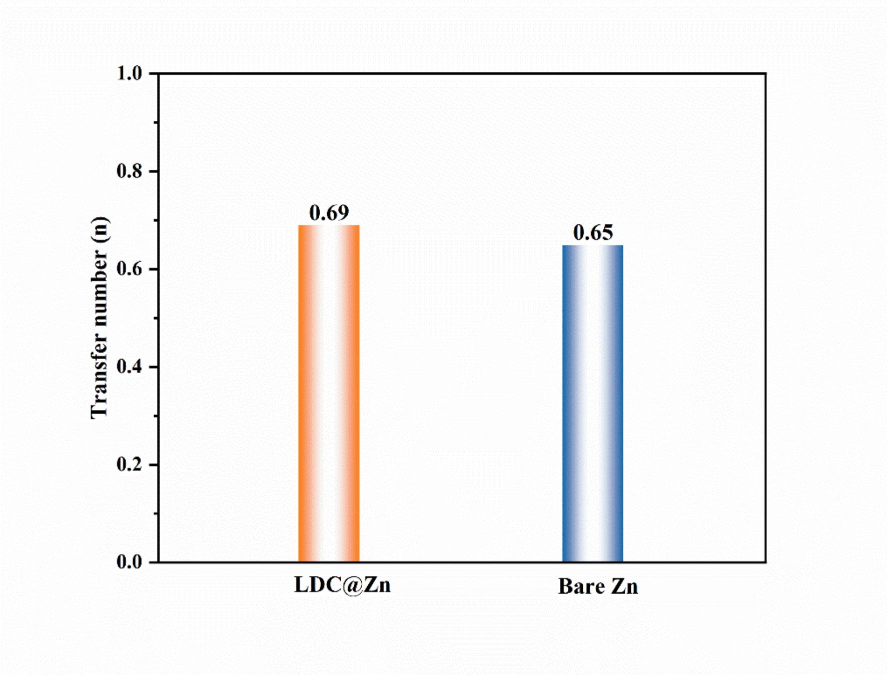

Figure S8. Zinc-ion transfer numbers of LDC@Zn and bare Zn.

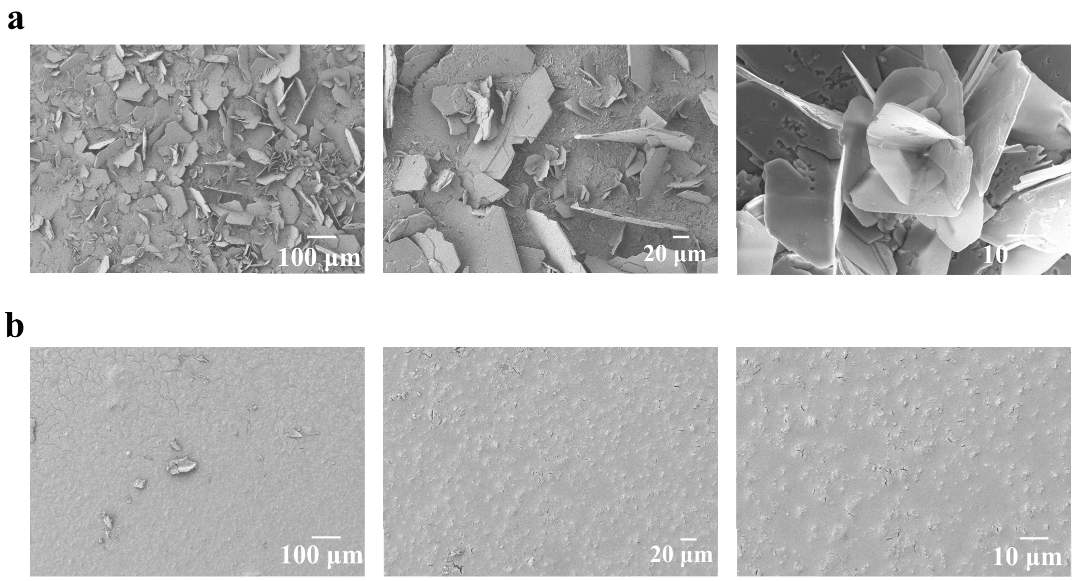

Figure S9. Top-view SEM images of the (a) bare Zn and (b) LDC@Zn

electrodes after immersion in 2M $ZnSO_4$ electrolyte for 10 days.

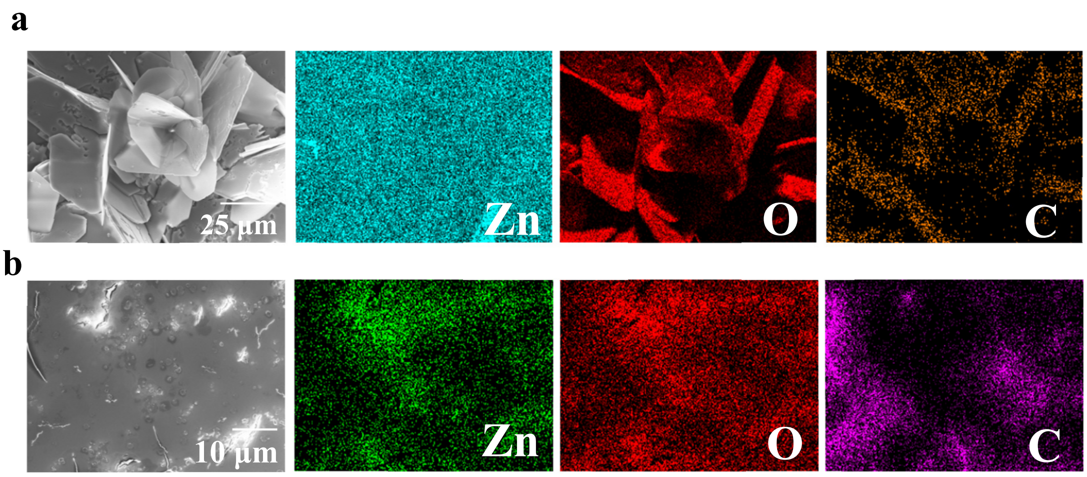

Figure S10. Corresponding EDS elemental mapping images of the (a) bare Zn and (b) LDC@Zn electrodes after immersion for 10 days.

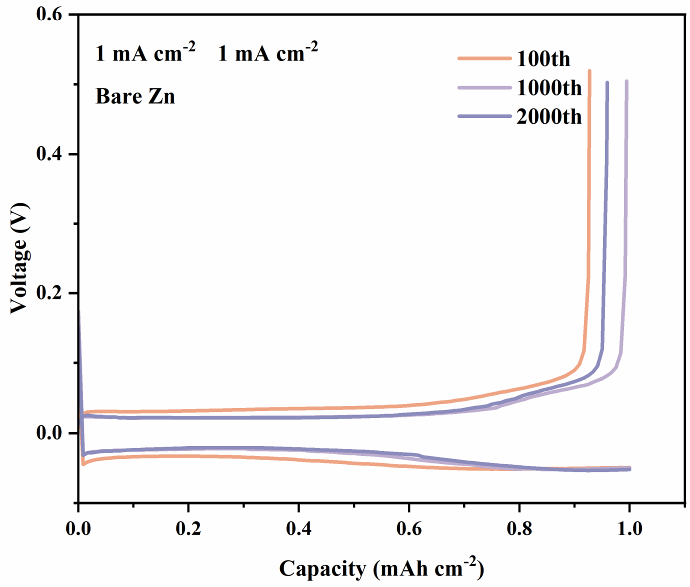

Figure S11. Voltage–capacity profiles of the bare Zn cell at the 100th,

1000th, and 2000th cycles at 1 mA cm^{-2} and 1 mAh cm^{-2}.

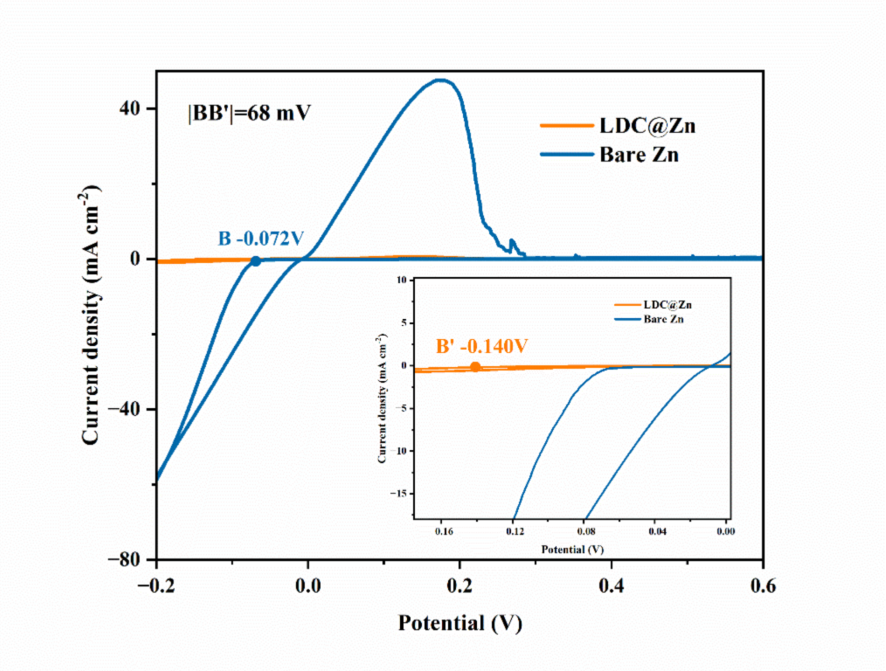

Figure S12. Cyclic voltammetry (CV) curves of the asymmetric cells.

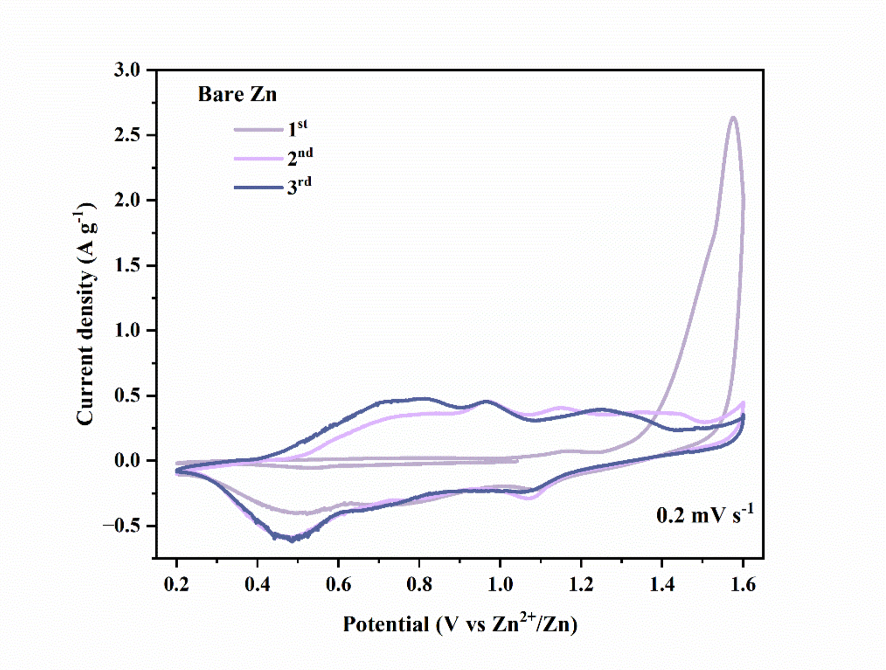

Figure S13. CV curves of the bare Zn full cell for the initial three cycles

at 0.2 mV s^{-1}.

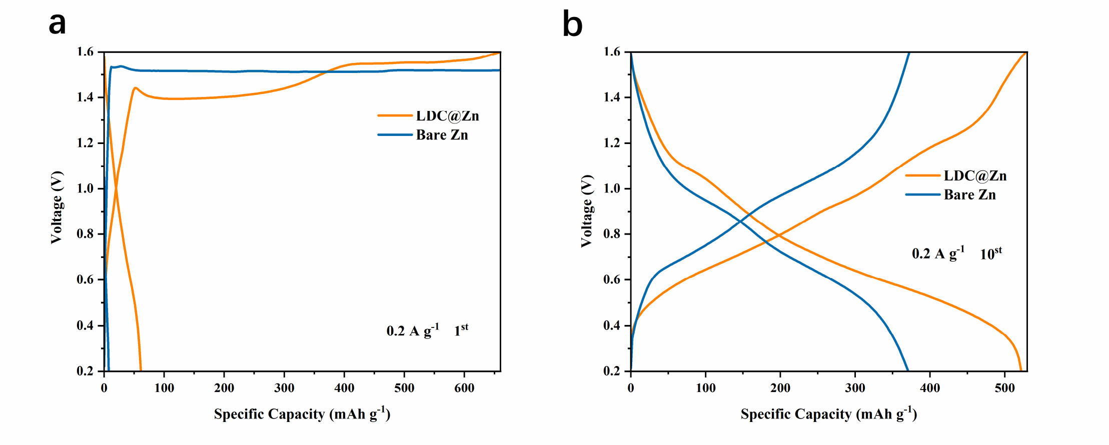

Figure S14. GCD profiles of the full cells with bare Zn and LDC@Zn anodes at the (a) 1st cycle and (b) 10th cycle at 0.2 A g^{-1}.
